# Let the two sides of the same coin meet—Environmental health and safety-oriented development of functional nanomaterials for environmental remediations

**DOI:** 10.1016/j.eehl.2024.06.001

**Published:** 2024-06-29

**Authors:** Shuangyu Wu, Jian Peng, Stephanie Ling Jie Lee, Xiaoqing Niu, Yue Jiang, Sijie Lin

**Affiliations:** a College of Environmental Science and Engineering, Biomedical Multidisciplinary Innovation Research Institute, Shanghai East Hospital, Tongji University, Shanghai 200092, China; b Key Laboratory of Yangtze River Water Environment, Shanghai Institute of Pollution Control and Ecological Security, Tongji University, Shanghai 200092, China

**Keywords:** Environmental health and safety, Functional nanomaterials, Pollution remediations, Safer-by-design, Sustainable-by-design

## Abstract

Nanotechnology and engineered nanomaterials have been at the forefront of technological breakthroughs of the 21st century. With the challenges of increasingly complex and emergent environmental pollution, nanotechnology offers exciting complementary approaches to achieve high efficiencies with low or green energy input. However, unknown and unintended hazardous effects and health risks associated with nanotechnology hinder its full-scale implementation. Therefore, the development of safer nanomaterials lies in the critical balance between the applications and implications of nanomaterials. To facilitate constructive dialogue between the two sides (*i.e.,* applications and implications) of the same coin, this review sets forth to summarize the current progress of the environmental applications of nanomaterials and establish the structure–property-functionality relationship. A systematic analysis of the structure–property-toxicity relationship is also provided to advocate the Safe and Sustainable-by-Design strategy for nanomaterials. Lastly, the review also discusses the future of artificial intelligence-assisted environmental health and safety-oriented development of nanomaterials.

## Highlights

• To facilitate constructive dialogue between the two sides (*i.e.*, applications and implications) of the same coin, the structure–property functionality and structure–property toxicity relationships of nanomaterials were established.
• Guided by the concept of life cycle analysis, the development of functional nanomaterials is evolving from safer-by-design to sustainable-by-design.
• Efforts to resolve the issue of data scarcity and incompatibility are required to forge meaningful integration between experimental and artificial intelligence-based tools to assist the investigation into environmental remediation.

## Graphical abstract

**Figure:**
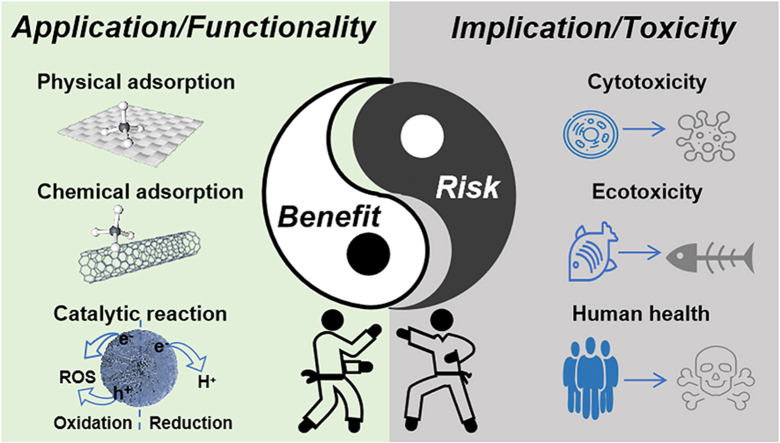

## 1 Introduction

Centuries of anthropogenic activities have continuously added burdens to the environment. As legacy and emerging pollutants possess both known and unknown toxic potential on living species, including humans, developing effective environmental remediation strategies has been one of the main focuses in securing ecological and public safety [1,2]. Over the years, technologies employing various physical, chemical, and biological processes have been able to remove conventional pollutants to a large extent [3]. However, conventional methods are unable to completely deal with newly produced chemicals and emerging compounds that are continually being discovered in the environment, which warrants the development of more effective technologies [4].

Nanotechnology has been at the forefront of creating novel solutions for environmental remediation. Pioneering work dating back to 1997 by Zhang et al. reported the first use of nano zero-valent iron (nZVI) for the in situ remediation of contaminated soils and groundwater [5]. Their ability to quickly and completely dechlorinate chlorinated aliphatic compounds and polychlorinated biphenyls stirred up excitement in the field. In addition to degrading pollutants through reduction, engineered nanomaterials also achieved oxidative removal of pollutants through catalytic reactions, including Fenton [6], Fenton-like [7], peroxymonosulfate activation [8], photocatalysis [9], etc. Evidenced by the photoelectrochemical water-splitting ability of TiO_2_, nanocatalysts with property tunability continued to be invented to target stubborn pollutants using reactive oxygen species (ROS) [10,11]. A quick search on “nanomaterials and environmental remediation” in literature resulted in more than 4000 publications, and this number continues to rise daily. However, the contrast between the ample literature and scarcity of real-life applications raises questions on what is preventing the full-scale application of nanomaterials and nanotechnology for environmental remediation.

Factors that determine the penetration of nanotechnology in environmental remediation include cost, ease of adaptability with existing technology, environmental health and safety (EHS) concerns, and public acceptance of nanomaterials [12,13]. Despite the advantages of reaching various environmental compartments, the properties of nanomaterials that provide the benefits of the nanoscale also lead to unintended environmental exposure and unknown EHS risks. This is probably one of the main reasons why there have only been about 70 cases of in-situ pilot studies using nZVI after its invention [14]. In 2006, Maynard et al. stressed the need to vigorously develop toxicity assessment tools for nanomaterials to narrow the knowledge gap [15]. Two decades of research revealed that nanomaterials can travel throughout the body, deposit in target organs, penetrate cell membranes, lodge in mitochondria, and trigger injurious responses [16]. As our ability to create new and unique nanomaterials advances exponentially, we should also take on the responsibility of understanding how these nanomaterials interact with biological systems [17]. In particular, how physicochemical characteristics of functional nanomaterials (i.e., size, shape, chemical composition, surface properties, aggregation and/or aggregation state, and biodegradability) affect their toxicokinetics (cellular uptake, transport, and responses) and toxicity mechanisms warrants careful study [18,19]. Although it is clear that data on the toxicity thresholds of nanomaterials would offer valuable knowledge to ease public fear, studies on the applications and implications of nanotechnology only intersect on a few occasions and the two sides of the same coin rarely meet [20].

Against this background, this review aims to provide a balanced perspective on the applications and implications of nanomaterials in environmental remediation. Keywords, including nanomaterials, environmental remediation/toxicity, and physicochemical characteristics, including size, functional group, exposed facets, etc., were used in the Web of Science search, and publications within the recent two decades were selected. To better understand this duality, the physicochemical properties of functional nanomaterials were taken as an anchor point to explore the structure–property-functionality and structure–property-toxicity relationship. Tuning the structures of nanomaterials can improve properties and enhance functions but may also cause unique adverse effects and toxicity mechanisms. Therefore, understanding the origin of toxicity can guide safer-by-design and manipulation of nanomaterial structures and properties at the synthesis stage to avoid adverse biological outcomes. Moreover, a broader framework that integrates the whole life cycle concept could provide a more comprehensive strategy for EHS-orientated development of nanotechnology, *i.e.,* sustainable-by-design. Lastly, the use of artificial intelligence (AI) tools to facilitate greener and sustainable development of nanotechnology is also discussed.

## 2 Structure–property-functionality relationships of nanomaterials

From the application perspective, the tuning of nanomaterials' properties leads to diverse functionalities. Current advances have showcased innovative approaches to adjusting characteristics such as size, functional group, facet, and redox capacity [[21], [22], [23], [24]]. Establishing relationships between these characteristics and corresponding functions could provide a list of design strategies for nanomaterials to accommodate various environmental remediation requirements.

### 2.1 Size and functional group

**Fig. 1:**
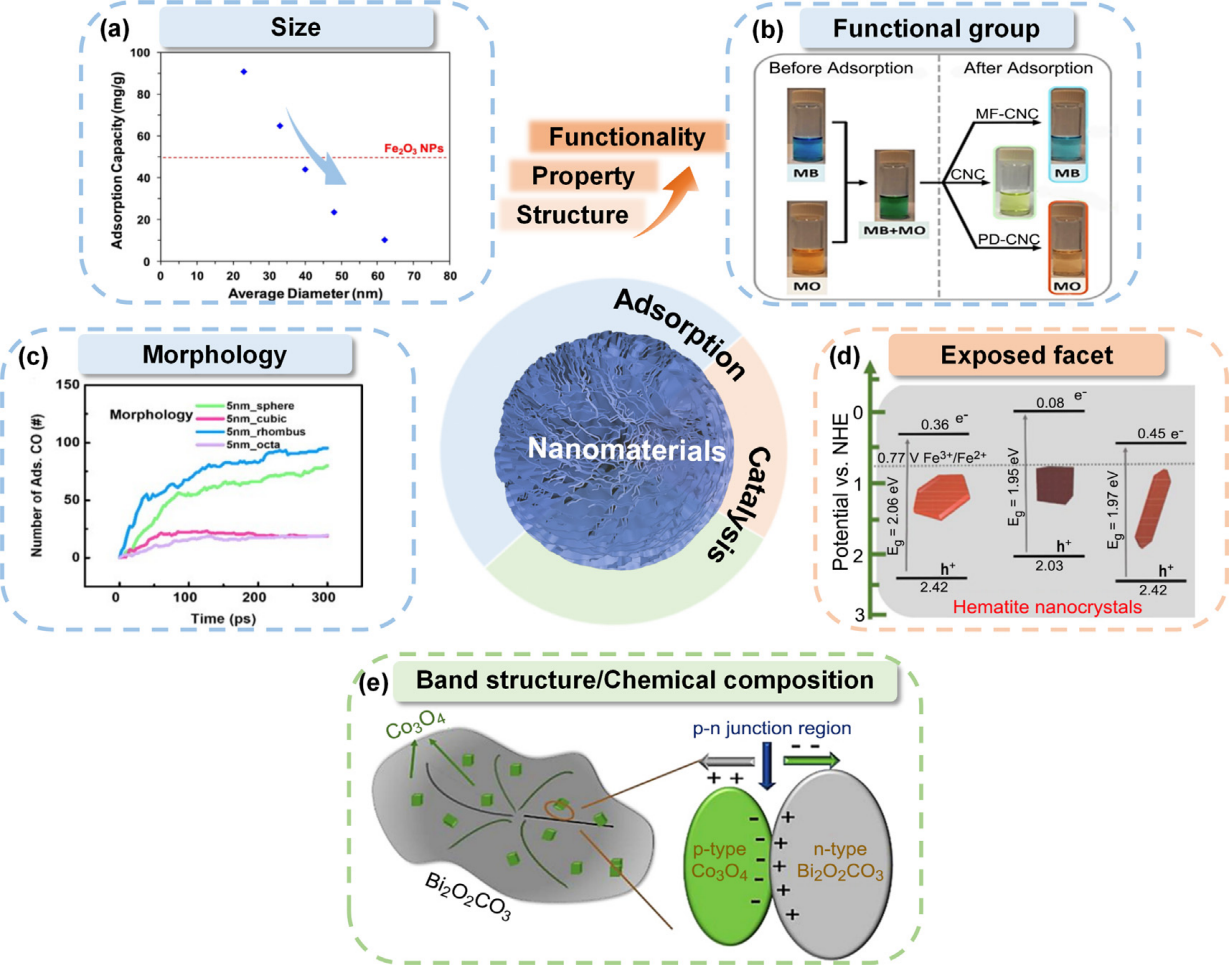
Structure–property-functionality relationships of nanomaterials. (a) The adsorption performance of Fe_2_O_3_ nanoparticles was enhanced with decreasing size. Reproduced with permission from ref [25]. Copyright (2015) Elsevier. (b) Selective adsorption and separation of methylene blue and methyl orange from the dye mixture were achieved by surface-functionalized cellulose nanocrystals. Reproduced with permission from ref [38]. Copyright (2021) Elsevier. (c) The amount of CO adsorption by Fe nanoparticles was affected by the variations of morphology. Reproduced with permission from ref [23]. Copyright (2019) Elsevier. (d) The hematite with the {001} facet produced free radicals more than the hematite with the {012} and {110} facets for contaminant degradation. Reproduced with permission from ref [46]. Copyright (2021) Elsevier. (e) The heterojunction construction increased the production of ROS for naphthalene degradation. Reproduced with permission from ref [61]. Copyright (2018) Elsevier.

Size determines specific surface area, hence the amount of adsorption, while functional groups determine adsorption specificity based on various adsorption mechanisms. Generally, smaller-sized nanomaterials, owing to their larger surface areas, provide more adsorption sites for pollutants, resulting in greater adsorption capacities. For instance, TiO_2_ with a size of 5 nm and a specific surface area of ∼248 m^2^/g, five times higher than that of 22 nm TiO_2_, possesses significantly greater adsorption capacity for organic compounds [21]. With decreasing particle size from 63 nm to 23 nm, Fe_2_O_3_ nanofibers exhibited superior chromate adsorption capacity due to their larger specific surface area (Fig. 1a) [25]. The adsorption of mercury from simulated flue gas by ZnS increased with increasing specific surface area (20.69, 120.70, and 144.35 m^2^/g) [26]. Biochar with the largest specific surface area was able to remove the most Cd(II), As(III), phosphate, and roxarsone from actual polluted soil among a range of different nano-goethite-modified biochar types [27]. These studies showcased ways to manipulate the size and specific surface area of nanomaterials by varying synthesis parameters [21,[28], [29], [30]]. For example, raising pyrolysis temperature within 450–650 °C for magnetic biocarbon resulted in higher specific surface area [28]; varying precursors resulted in smaller size and higher specific surface area of zeolitic imidazolate framework-8 (ZIF-8) crystals [31]; introducing β-cyclodextrin could control crystal growth to yield nano-sized zeolite [32].

Functional groups enable specific adsorption of pollutants either based on binding affinities or by introducing electrostatic/steric hindrance to avoid unwanted adsorption. Manipulation of surface functional groups was typically achieved by replacing precursors with functional group components or by grafting surface-modifying reagents onto the material's surface [33]. The influence of surface functional groups on adsorption was mainly related to ion exchange, electrostatic interaction, surface complexation, hydrogen bonding, π-π interaction, etc [22,34,35]. For instance, graphene oxide with oxygen-containing functional groups fostered electrostatic attraction, hydrogen bonding, and complexation interactions with Cu ions, leading to greater Cu ions removal [36]. Amination of metal–organic framework (MOF) resulted in high adsorption capacity for uranium in natural seawater environments via hydrogen bonding while avoiding the adsorption of other molecules or cations [37]. Cellulose nanocrystals modified with sulfate ester groups and amine groups were able to separate methylene blue and methyl orange from mixtures due to preferential adsorption (Fig. 1b) [38]. Similarly, Br-modified zirconium-based MOF (UIO-66-Br_2_) with the polarizability of the Br functional group adsorbed CH_4_ while rejecting N_2_ to achieve separation and purification of mixed gas [39]. These approaches also have the potential to achieve seawater desalination and separation of alcoholic water at room temperature [40].

### 2.2 Exposed facet

For crystalline nanomaterials, alterations of size and morphology were often accompanied by changes in exposed facets [30,41]. In this case, the adsorption characteristics were also determined by the exposed facets besides specific surface area. For example, the amount of CO adsorbed by Fe nanoparticles of different crystallinities showed the following trend, rhombus > sphere > octa > cubic (Fig. 1c), in which rhombus Fe nanoparticles were composed of pure {110} facets [23]. α-Fe_2_O_3_ of different facets showed different maximum adsorption of Pb(II) ion, Cd(II) ion, and Hg(II) ion at {001}, {116}, and {110} facets, respectively. The adsorption energy (E_ad_) generated when a substance was adsorbed to one particular exposed facet served as the main characteristic of the specific adsorption, and the greater the E_ad_, the greater the adsorption affinity [42,43]. Rutile TiO_2_ with {110} facets had the greatest adsorption capacity for 1,1,2,2-tetrachloroethane than TiO_2_ with other crystalline phases because of its strongest adsorption affinity [44]. Exposed facets of nanomaterials have also shown specific catalytic reactivities to contaminants. The exposed facets of iron (hydr)oxide nanoparticles determined the adsorption affinity and catalytic activity toward 4-nitrophenyl phosphate [45]. The {001} facet of hematite activated persulfate molecules to produce free radicals more than the {012} and {110} facets (Fig. 1d) [46]. MnO_2_ of {310} facets, which possessed higher surface energy than other facets ({110} and {100}), showed better catalytic ozonation of CH_3_SH [47]. The oxygen vacancy of the {310} facets also facilitated the adsorption and activation of O_3_ into intermediate peroxide species (O^2−^/O_2_^2−^) and ROS (•O_2_^−^/^1^O_2_) to eliminate adjacent CH_3_SH. Strategies to control the exposed facets were typically achieved by modulating the surface energy of the nanomaterials [48]. Examples included the use of different capping agents to reduce the surface energy of specific facets, resulting in the exposure of specific facets, the adjustment of pH or temperature to control the growth of specific facets, and the control of reaction time to preserve specific facets [[49], [50], [51], [52]].

### 2.3 Redox reactivity

In pollution remediation, adsorption only transfers contaminants from one phase to another. To achieve effective removal or degradation, adsorption is often accompanied by catalytic reactions that involve the breaking of chemical bonds and structures. The catalytic reactivity of functional nanomaterials is largely affected by their redox capacity, which refers to the ability to produce highly reactive species through electron transfer in response to external stimuli (light, electricity, etc.) [53,54]. In reduction reactions, the formation of reducing species (e.g., photogenerated electrons, reactive atomic hydrogens) was the main factor controlling the degradation efficacies of pollutants. Starch-stabilized Fe–Pd nanoparticles showed greater dechlorination capacity toward chlorinated hydrocarbons than that of non-stabilized nanoparticles due to better dispersion and greater generation of active atomic hydrogen [55]. Construction of surface defects also proved to be effective in promoting the generation of reducing species. Surface defects of ZnO worked as photogenerated hole trappers, retaining effective photogenerated electrons to achieve photoreduction of Cr(VI) [56]. The addition of the metal Pb regulated the band structure of Fe nanoparticles, leading to the promotion of electron transfer and enhanced production of reactive atomic hydrogen [57]. Similarly, the addition of transition metals (Ni and Cu) to metal nanoparticles could also significantly accelerate the reduction degradation of organic halogen compounds [14,58]. Moreover, heterostructures formed by nZVI, g-C_3_N_4_, and MoS_2_ facilitated the separation of photogenerated electron–hole pairs for superior catalytic properties in the reduction reactions of Cr(VI), Pb(II), and Cd(II) [59].

In oxidation reactions, the formation of oxidizing species (e.g., •OH, •O_2_^−^, ^1^O_2_) is the main player controlling the efficacies of pollutant degradation and mineralization [60]. Compared with pure TiO_2_, elemental (C and N) doping narrowed the bandgap of TiO_2_ and significantly improved the migration efficiency of the carriers to produce more ROS for tetracycline removal [9]. Constructing heterojunctions also showed effectiveness in promoting ROS generation. Compared with single-component nanocatalyst (e.g., Co_3_O_4_), a type p-n heterojunction formed between Co_3_O_4_ and Bi_2_O_2_CO_3_ optimized the separation of electron–hole pairs, resulting in greater naphthalene degradation (Fig. 1e) [61]. A double Z-type heterojunction formed by InVO_4_, CuBi_2_O_4,_ and BiVO_4_ allowed the photoinduced electron to flow from the conduction band (CB) of InVO_4_ and BiVO_4_ to the neighboring valence band (VB) of CuBi_2_O_4_. As a result, the hole on VB of InVO_4_ and BiVO_4_ and the electron on CB of CuBi_2_O_4_ with stronger redox capacity were preserved [62]. Moreover, by converting Ti-deficit vacancies of the exfoliated Ti_3_C_2_ MXene into discrete titanium oxide with multivalent Ti, the highly-confined nanoclusters showed much greater ROS generation capability [7]. With a further decrease of size to the single-atom scale, single Ru atom catalysts (Ru–N–C) showed superior activation of peroxymonosulfate for Orange-II degradation [63]. In short, strategies to manipulate the redox capacity include minimizing particle agglomeration, element doping, constructions of surface defects, heterojunctions, nanoclusters, and single-atom catalyst formations [55,56,[64], [65], [66]]. As chemical compositions of nanomaterials were affected by element doping and the construction of heterojunctions, they also showed a close correlation with the redox capacity.

The delineation of the structure–property-functionality relationship shows that efforts have been made to manipulate the physicochemical characteristics of the as-synthesized nanomaterials to enhance performance in the course of investigating environmental applications. When functionalities were the sole goal, research overwhelmingly focused on the pursuit of efficacies, which blinds us from seeing the risks associated with nanomaterials. The explosive growth of nanomaterials would lead to inevitable exposure to the environment and living species. With that comes concern about their risks in the realm of environmental health and safety.

## 3 Structure–property-toxicity relationships of nanomaterials

While counterintuitive, nanomaterial toxicology analysis is closely related to its functional exploration as both function and toxicity are often derived from the same characteristics. Physicochemical characteristics of nanomaterials, including size, aspect ratio, shape, surface functional groups, hydrophobicity, surface charge, crystallinity, band structure, and chemical composition [67,68], determine their surface properties which influence intimate nano-bio interfacial interactions governing the toxicological outcomes exerted by nanomaterials [67]. From the point of view of risks associated with nanomaterials, toxicologists aim to establish structure–activity relationships between the physicochemical characteristics and toxicity of nanomaterials using *in vitro* and *in vivo* models to predict human health and environmental hazard potential. With that, reduction of toxicity becomes feasible by tuning physicochemical characteristics to improve biocompatibility and obtain safer and greener nanomaterials.

### 3.1 Size, aspect ratio, and shape

**Fig. 2:**
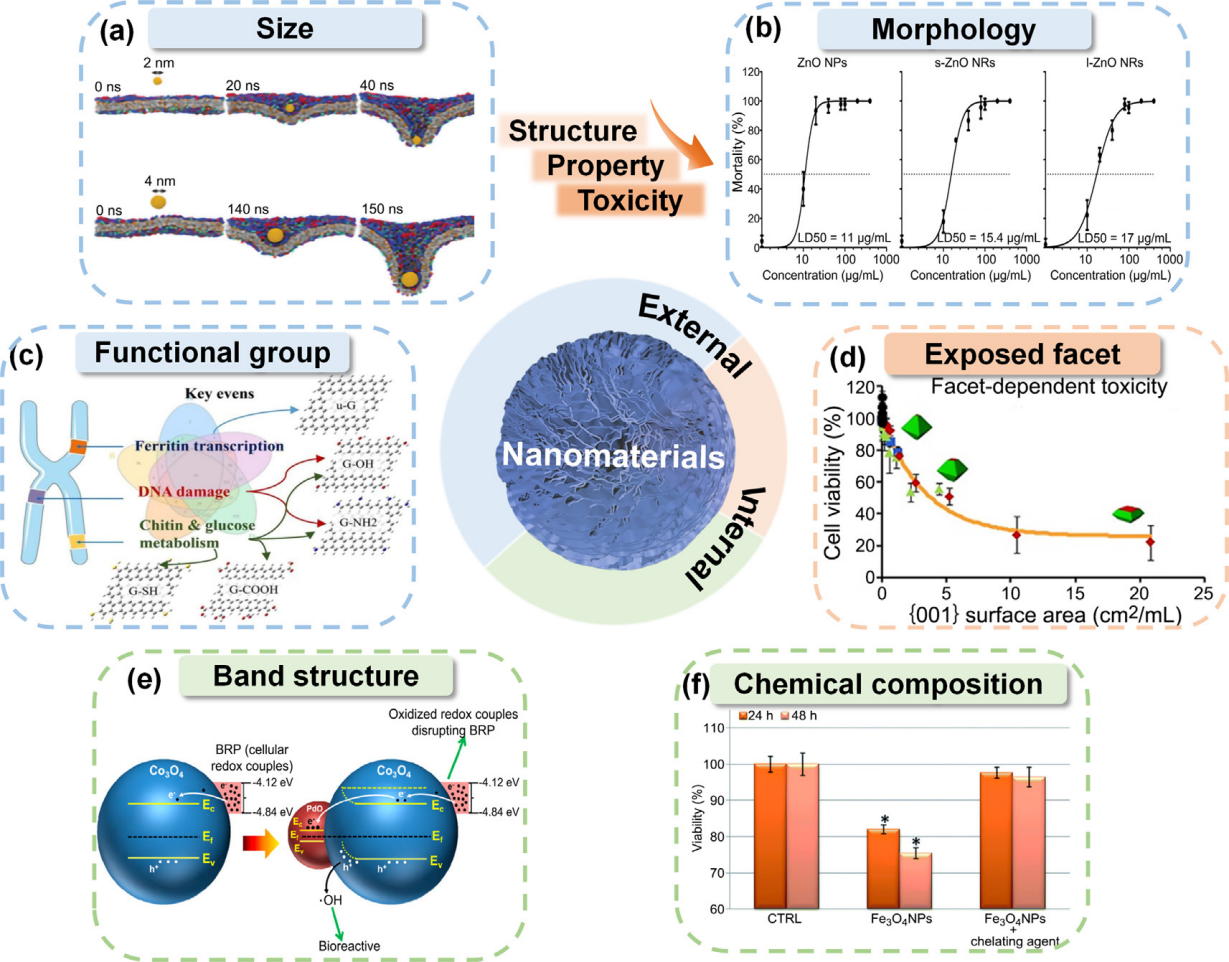
Structure–property-toxicity relationships of nanomaterials. (a) Spherical Au nanoparticles showed pronounced size dependence in their translocation through the plasma membrane. Spherical Au nanoparticles are shown in yellow. Reproduced with permission from ref [70]. Copyright (2019) American Chemical Society. (b)The LD_50_ of ZnO nanomaterials in zebrafish larvae is related to morphologies of ZnO nanomaterials. Reproduced with permission from ref [85]. Copyright (2021) Elsevier. (c) Different surface functional groups of graphene can alter their chronic toxic mechanisms to *Daphnia magna*. Reproduced with permission from ref [89] Copyright (2023) Elsevier. (d) Cell viability decreased with the increase of titanium dioxide {001} facet percentage. Reproduced with permission from ref [110]. Copyright (2016) American Chemical Society. (e) The level of oxidative stress of cells induced by Co_3_O_4_ as a result of Ec overlaps with the BRP could be accentuated by PdO doping. Reproduced with permission from ref [115]. (f) The general mechanism of toxicity caused by metal ions released by nanoparticles when they enter cells. Reproduced with permission from ref [118].

Nanomaterial size, aspect ratio, and shape dictate protein adsorption, cellular uptake, transport, and biological reactivity, which ultimately determine the hazard potential [69]. Gold nanoparticles showed pronounced size dependence in their translocation through the plasma membrane (Fig. 2a) [70]. Gold nanoparticles with diameters less than 10 nm were shown to directly penetrate the membrane bilipid layer through passive diffusion. Cellular uptake of larger nanomaterials proceeded via direct penetration of the cell membrane [71] or energy-dependent endocytosis-mediated uptake pathways [68]. A nanoparticle threshold radius existed where wrapping time was faster for nanoparticles with a larger radius below this optimal particle radius (*r*_opt_). However, wrapping time increased beyond this *r*_opt_ due to slower receptor diffusion kinetics [67]. Cellular uptake of smaller nanomaterials was generally faster than for larger nanomaterials, with 50 nm identified as the optimal size for receptor-mediated endocytosis [72,73]. Greater ATPase activity inhibition and Na^+^/K^+^ channel disruption at the basolateral membrane of adult zebrafish gills and intestine were observed for 20 nm citrate-coated silver nanoparticles compared to 110 nm nanoparticles [74]. While larger nanomaterials contributed to cytotoxicity by disruption of membrane integrity through pore formation in the cell membrane [75], smaller nanoparticles were more detrimental in causing oxidative stress [76]. In general, a size of 30 nm and below was considered a critical size threshold for exerting adverse biological effects [77].

There is a nanomaterial-specific optimal aspect ratio for nanomaterial cellular uptake. The cellular uptake of gold nanoparticles with a smaller aspect ratio was faster than for gold nanoparticles with a larger aspect ratio. This was attributed to the longer membrane wrapping time required for nanoparticles with larger aspect ratios [68]. A larger aspect ratio is linked with elevated toxic potential [78,79]. At aspect ratio ≥22, CeO_2_ nanorods triggered lysosomal damage, NLRP3 inflammatory responses, and cytotoxicity in the human myeloid cell line (THP-1) [78]. *In vitro* studies showed that clearance of high aspect ratio nanomaterials, e.g.*,* long multi-walled carbon nanotubes [80] and TiO_2_ nanobelts [81], triggered frustrated phagocytosis and lysosomal damage, induced pro-inflammatory responses, and increased cytotoxicity in macrophage cells.

The shape also influences the bioavailability and *in vivo* toxicity of nanomaterials due to the degree of curvature. Nanomaterials with a higher degree of curvature, such as spherical ones, generally enter cells more readily than rod-shaped nanoparticles [72,73]. The cellular uptake rate of spherical gold nanoparticles was 5–7 times that of rod-like nanoparticles [82]. Molecular simulations have shown that spherical nanoparticles have the lowest membrane-bending energy during endocytosis and, therefore, possess the fastest endocytosis rate [83,84]. When the toxic effects of the three ZnO shapes (nanospheres, short and long nanorods) were compared, the LD_50_ of ZnO nanospheres was slightly lower than that of ZnO short and long nanorods (Fig. 2b) [85]. This suggests that the higher toxicity of spherical nanoparticles was partly due to their ease of internalization. Overall, spherical particles are taken up preferentially by the cells, as their geometry requires making fewer contacts with cell surface receptors while also requiring less free energy for particle wrapping. In short, size, aspect ratio, and shape influenced membrane wrapping, a key determinant of cellular uptake and its rate.

### 3.2 Functional group and exposed facet

Interactions between the nanomaterial and various cells and/or biomolecules can be advantageous, neutral, or toxic depending on its functional groups [86]. Functional group modification can change the surface hydrophobicity, surface charge, and charge density of nanomaterials, which affect protein adsorption, cellular uptake, and biological fate [[87], [88], [89]]. Different functional groups of nanomaterials can alter their toxic mechanisms in organisms (Fig. 2c) [89]. The relative surface hydrophobicity/hydrophilicity of the nanoparticle influences protein adsorption, cellular permeability, cellular uptake, biological fate, and toxicity. Upon contact with a biological fluid (e.g., blood or plasma), nanomaterials can bind to proteins to form a nanoparticle-protein corona [67]. Plasma proteins control the cellular uptake and biological fate of nanoparticles since they are linked to phagocytosis [90]. Protein adsorption was greater for hydrophobic nanomaterials than hydrophilic ones [91]. Hydrophobic gold nanoparticles were internalized more quickly and induced greater cytotoxicity to THP-1 macrophage cells than hydrophilic gold nanoparticles [92]. Simulations using the synthetic supported lipid bilayer (SLB) membrane showed that semi-hydrophobic carboxyl-functionalized polystyrene nanoparticles adsorbed to the SLB, drawing lipids away and creating lipid-poor regions ‘pores’ in the SLB, thereby increasing membrane permeability [93]. In addition, hydrophobic gold nanoparticles generated ROS in HeLa cells via NADPH oxidase activation [94].

Surface charge and charge density also influence protein adsorption, cellular uptake rate, and membrane integrity. Adsorption of plasma proteins increased with surface charge density [95]. Cationic particles attach to the cell surface more easily than neutral or anionic nanoparticles due to electrostatic interactions with the negatively charged membrane bilipid layer [96]. The positively charged nanoparticles can disrupt the cell membrane potential and ultimately damage the lipid bilayer [97]. In addition, high cation density can lead to physical membrane damage through pore formation, membrane thinning or erosion, and/or expansion of pre-existing membrane defects [[97], [98], [99], [100], [101]], which is associated with increased cellular uptake [97,102,103], leakage of cytosolic enzymes across the cell membrane [99,100], mitochondrial damage [104], increased intracellular calcium flux [94,97,104] and increased intracellular ROS production [94,104]. Cationic nanoparticles also caused lysosomal damage via the proton sponge effect, leading to cytotoxicity [105]. In addition, negatively charged surface silanol (SiO^−^) groups of mesoporous silica nanoparticles formed hydrogen bonds with the cell membrane through electrostatic interactions, disrupting cell membrane structure and hence contributing to toxicity [106]. Generally, the higher the charge density, the greater the toxic potential [107].

Exposed facets of nanomaterials determined the adsorption of proteins on nanomaterials by surface complexation, which may also alter the toxicity of the nanomaterials [108]. Exposed facets with high surface reactivity catalyzed ROS generation to induce significant toxicity. ZnO nanoneedles (ZnO-1010) with primary exposed {1010} facets exhibited greater cytotoxicity towards Caco-2 cells than ZnO nanoflakes (ZnO-001) with primary exposed {0001} facets [109]. The vacancy formation energy (1.15 eV) of the {1010} facet of ZnO-1010 was lower than the {0001} facet (3.90 eV) of ZnO-001, resulting in greater dissolution of ZnO-1010 to Zn_3_(PO_4_)_2_ and •O_2_^−^generation unlike ZnO-001 which produced O_2_^2−^, resulting in oxidative stress and membrane damage for ZnO-1010 [109]. The results showed that the formation of ROS was affected by different exposed facets of ZnO nanoparticles, which resulted in facet-dependent toxicity. Another study also corroborated the increased production of •OH on the {001} facets of TiO_2_ nanocrystals compared to {101} facets, which endowed {001} facets with strong hemolytic activity and elicited severe toxicity (Fig. 2d) [110]. Therefore, for nanomaterials with abundant active sites on exposed facets, the type and proportion of facets determine the final amount of ROS production, resulting in different degrees of biological damage.

### 3.3 Band structure and chemical composition

Under normal physiological conditions, the redox equilibrium is maintained in the mitochondria of biological systems through low levels of ROS production balanced by enzymatic and non-enzymatic antioxidants such as superoxide dismutase and glutathione. The two main physicochemical characteristics of nanomaterials that confer redox reactivity include: (1) overlap of band gap with cellular redox potential, and (2) metal ion release. Band gap determines ROS production and oxidative stress potential. Electron transfer can occur when the relative energetics of the top of the valence band and bottom of the conduction band of the metal oxide semiconductor nanoparticle overlap with the cellular redox potential (E^0^) [[111], [112], [113]]. Mammalian cell line and *Escherichia coli* studies have shown that this disrupts cellular redox homeostasis and increases oxidative stress through elevated production of oxidizing or reducing substances that reduce antioxidant levels and increase ROS and/or oxidized biological component levels, leading to growth inhibition and increased cellular mortality [112,113]. Nanoparticle doping can alter the nanoparticle oxidative stress and hazard potential by modifying the band structure [114]. Electron transport between the biological redox pair and conduction band was faster for PbO-doped Co_3_O_4_ nanoparticles than pristine Co_3_O_4_ nanoparticles, subsequently producing more extracellular •OH (Fig. 2e) [115]. The greater the overlap between the band structure of nanomaterials and the redox potential of the biological system, the greater the levels of extracellular ROS produced and the more severe the resulting toxicity. Therefore, the difference between the material band gap and cellular redox potential (from −4.2 to −4.8 eV) can be used to predict the oxidative stress potential of the metal oxide nanoparticle [112].

The presence of different metals in nanoparticles also affects the physicochemical properties and toxicity of metal oxide nanomaterials due to different solubilities. Dissolution and metal ion shedding are the key contributing factors that lead to toxicity for soluble metal and metal oxide-based nanomaterials. For extracellular metal ion shedding into the surrounding medium, nanoparticles typically attach to the cell membrane and interact with surface proteins, culminating in compromised stability of the cell membrane. For instance, Ag ions released by Ag nanoparticles could trigger apoptosis when adhering to cells, and the greater the number of Ag ions released, the higher the level of apoptosis [116]. In the “Trojan horse” scenario, nanomaterials are internalized by cells as particles, only to deliver toxic chemicals inside the cell after entry [117]. For example, the considerable amount of intracellularly leaked ions exerted ion-specific toxicity against cellular targets (e.g., mitochondria) and lysosomal damage/dysfunction (Fig. 2f) [118]. Compared to practically insoluble CeO_2_ and TiO_2_, ZnO dissociation induced intracellular ROS production, resulting in apoptosis [105]. The extent of biological damage is linked to the quantity of ion leakage. It was found that transition metal oxides (such as Mn_2_O_3_, CuO, and ZnO) and rare earth oxides (such as Gd_2_O_3_, La_2_O_3_, and Y_2_O_3_) were the most toxic metal oxides in hepatocytes due to their high solubility among 29 oxides nanoparticles [119]. In general, the greater the number of metal ions released by nanomaterials, the more ROS and oxidative stress they generate, and the greater the cellular damage they cause.

Given the above discussion on the structure-properties-toxicity relationships of nanomaterials, the importance of establishing the link between the physicochemical properties of nanomaterials and their toxicity is self-evident. If the scientific community provides a reproducible link between specific nanomaterial features and a particular biological response, less toxic or green nanomaterials might then result from “designing-out” features identified to cause toxicity.

## 4 EHS-oriented development of nanomaterials

Advances in nanotoxicology serve as reminders that the remarkable physicochemical properties of nanomaterials could lead to significant environmental health and risk, thereby influencing their application. Beyond establishing regulatory frameworks for safer implementation of nanotechnology, insights on toxicology can be extrapolated to better predict the hazard potential, determine toxicological mechanisms, and guide the design of nanomaterials. Safer-by-design approaches have emerged over the years and applied to nanomaterial design, especially in the field of nanomedicine [120,121]. While the safer-by-design approach mainly focuses on ensuring safety at the synthesis stage, sustainable-by-design, which takes into account the migration, transformation, and resource utilization efficiency throughout the entire life cycle of nanomaterials, could provide a more comprehensive strategy to facilitate further development of sustainable nanotechnology.

### 4.1 Safer-by-design

The “Safer-by-design” strategy aims to create less toxic and more environmentally friendly materials at the design phase of innovation, sharing a similar mindset of green chemistry [122,123]. Consistently, the European Union initiated a series of safe-and-sustainable-by-design (SSbD) chemical strategies with the objective of enhancing the safety and sustainability of chemicals in 2019 [124]. The establishment of SSbD strategies augmented the need for safer-by-design of nanomaterials to minimize the risks associated with their fabrication and application phases. Nanomaterials possess a wide range of characteristics that can be designed or tuned, depending on requirements, to enhance functionality or reduce toxicity. The previous sections provide a solid foundation on structure–property-functionality and structure–property-toxicity relationships of engineered nanomaterials. Addressing specific toxicity has become pivotal to promoting the application of nanomaterials, which has led to widespread adoption of the “safer-by-design” approach for nanomaterial development [120,121].

The external properties of nanomaterials, such as surface charges, functional groups, and other surface properties, can be engineered by surface modification to make their application safer. By modifying carboxyl groups, the surface charge of nanoparticles can be changed from positive to negative, which can significantly reduce the production of the inflammatory factor TNF-α in human cells. Similarly, modification of polyethylene glycol (PEG) on the surface of CuO nanoparticles can inhibit protein adsorption and thus inhibit the uptake of nanoparticles by macrophages [125]. Silica nanoparticles with almost free silanol groups disrupt cellular membranes, whereas modulation of the silanol groups by the flame spraying process can effectively control cytotoxicity. In addition, carbon nanotubes can be made biologically safer by modifying them on hydrophobic surfaces [126]. By depositing β-lactoglobulin (a whey protein) amyloid-like fragments (ba) onto the surface of carbon nanotubes (baCNTs), the functionalized CNTs could trap toxic human islet amyloid polypeptide (IAPP) species thereby reducing IAPP amyloidogenesis *in vivo* [127].

The interior properties of nanomaterials, such as the internal release of specific elemental constituents and band structure, lead to hazardous effects that can be mitigated by element doping and regulation of Fermi levels. For example, the ion leaching of Ag and ZnO nanoparticles is reduced by tellurium doping and iron doping to achieve a safer antibacterial effect [114,128]. Doping of transition metal elements tuned the E_c_ value of Mn_3_O_4_ nanoparticles away from the biological redox potential range (BRPR), avoiding toxicity caused by electron transfer between nanoparticles and biological systems [111]. Furthermore, the Fermi energy of Mn_3_O_4_ nanoparticles can also be adjusted by transition metal doping to lower hole generations and oxidative damage [129]. Iron doping of ZnO was also used successfully to lower ZnO nanoparticle dissolution and reduce toxicity in human bronchial epithelial and macrophage cell lines [130], rodent lungs, and zebrafish embryos [114].

In addition to the above examples, a study showed that superparamagnetic iron oxide nanoparticles synthesized using green tea extract as a reducing agent exhibited both greater removal capability toward malachite green and significantly lowered toxicity [131]. However, examples showcasing improvements in both functionality and biocompatibility were still rare due to the disconnect between different fields. Moreover, even if the structural features of nanomaterials that may cause toxicity are circumvented at the design stage, the characteristics of nanomaterials may change due to environmental influences during usage [132,133]. Therefore, the effects of environmental factors (such as humic acid, soil pH, anions, and cations) on the properties of nanomaterials should also be considered during applications [134]. For example, nanomaterials used for in situ remediation could cause unexpected hazards. The use of nZVI in situ remediation of Cr(VI) contaminated soils bears the potential risk of introducing Cr(VI) into groundwater systems [135]. The concept of making nanomaterials safer should not be limited to the design phase but also extend throughout the full life cycle, i.e., from “cradle” to “grave”.

### 4.2 Sustainable-by-design

**Fig. 3:**
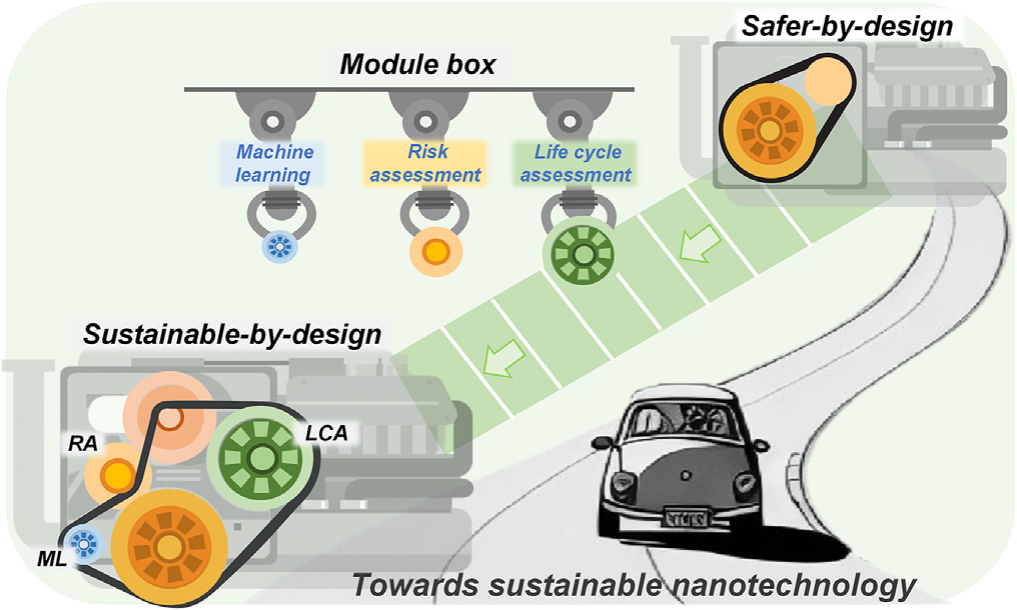
The road for sustainable-by-design of nanomaterials.

“Sustainable-by-design” builds upon the foundation of “safer-by-design” and addresses the sustainability aspects of materials/products and their associated processes throughout the entire life cycle [136]. In June 2022, the EU revised the definition of nanomaterials in order to establish a coherent regulatory framework for nanomaterials based on the previous SSbD chemical strategies [124]. This concept extends to the field of nanotechnology, which not only optimizes the safe implementation of new nanomaterials but also emphasizes safety and sustainability throughout the full life cycle. Currently, assessment methods, including life cycle assessment (LCA) and risk assessment (RA), and design tools, such as machine learning (ML), could be employed to facilitate the development of more sustainable nanomaterials (Fig. 3).

LCA can be used to compile and evaluate the inputs, outputs, and potential environmental impacts of products throughout their entire life cycle, providing a valuable reference for sustainable-by-design approaches [137]. Regarding the production of nanomaterials, LCA quantitatively compared the environmental impacts and costs of the green synthesis method for the fabrication of nZVI from ecologically sourced materials versus the traditional borohydride synthesis method [138]. The green synthesis route produced a greater than 50% reduction in environmental impact and an eightfold cost reduction compared to traditional approaches. LCA showed that consumption of ethanol and electricity exerted the greatest environmental impact during the synthesis of TiO_2_/FeCl_3_. Hence, subsequent design of TiO_2_/FeCl_3_ materials should aim to minimize the use of ethanol and electricity [139]. During nanomaterial utilization, LCA analyzed the environmental burden generated by different radiation sources (solar, visible light, ultraviolet-A) on the photocatalytic reaction of the nanocatalyst (TiO_2_/FeCl_3_) and concluded that solar-driven photocatalysis had the lowest environmental impact [139]. LCA has evolved as a pivotal aspect of sustainable design, and the fusion of specific nano impacts and LCA environmental impact categories, such as human toxicity and ecological toxicity, could further propel the advancement of sustainable nanomaterial design.

RA enables the quantification of potential exposure and hazards associated with nanomaterials under various release scenarios, filling in the gaps of LCA ecotoxicity considerations [140]. To better integrate RA with LCA, a series of strategies and tools such as multi-criteria decision analysis (MCDA) and Ashby materials selection framework has been developed to more effectively reveal the inherent complex trade-offs [141]. Ashby materials selection framework and other assessment methods combined the decision criteria derived from RA and LCA results with non-environmental criteria, such as cost and material performance, to form a basis for holistic sustainability analysis [142]. A modified framework based on Ashby's material selection strategy also takes cumulative energy demand, water usage, greenhouse gas emissions, and other environmental life cycle impacts into account [136]. Moreover, it includes specific indicators for environmental/human health impacts to construct two-dimensional or three-dimensional tables. This approach has proven effective in the design of nanomaterials [141]. The combination of LCA and RA has a positive and beneficial effect on advancing the sustainable design of nanomaterials. With the exponential growth of nanomaterials, achieving sustainable-by-design requires extensive and efficient data integration and individual comparisons [143].

ML could be an effective tool to accelerate the development of “sustainable-by-design” nanomaterials by enhancing efficiency and obtaining more effective data when combined with LCA or RA [144,145]. During the synthesis stage, the use of ML can optimize nanomaterial synthesis methodology, which is more efficient than traditional trial-and-error methods [146]. For instance, the researchers can maximize the sustainability of ZIF-8 synthesis by employing non-dominated sorting genetic algorithm II (NSGA-II) [147]. The method recommended by the algorithm can rapidly and accurately obtain ZIF-8 with 100% yield and 88% crystallinity, significantly reducing the carbon footprint by more than 40%. ML enables effective toxicity prediction while improving data collection efficiency. Deep-learning-based morphometric analysis can quantitatively identify various abnormal phenotypes of zebrafish larvae in toxicity experiments, with a single image recognition time of 0.075 s, facilitating rapid and accurate morphological analysis and minimizing the collection of inaccurate data arising from interindividual differences in human judgment [148]. Through data training, it can predict the biological pathways involved and identify potential toxicity mechanisms, thus facilitating the discovery of meaningful information embedded within large datasets.

## 5 Summary and outlook

It has been recognized that the functions of nanomaterials can be maximized through the manipulation of structures and properties. At the same time, developments in nanotoxicology have raised concerns about the environmental exposure and health implications of nanomaterials. Research on applications and implications of nanomaterials have mostly been carried out in isolation from each other thus far. This situation impedes progress at best and leads to misleading reports that fuel public fear and mistrust of nanotechnology at worst. Thus, research into nanomaterials applications and implications should be coordinated. Furthermore, despite some pioneering work using machine learning tools to design safer nanomaterials and predict their toxicity, many challenges remain for the EHS-oriented development of functional nanomaterials.

The first challenge is data scarcity. For example, LCA on nanomaterials lacks data on the final disposal stage, mainly because the environmental remediation field does not pay attention to this stage. In addition, the speed of RA of nanomaterials cannot keep up with the speed of its iterative update, and a large amount of toxicity data of new nanomaterials are missing. For ML, its training set requires a huge amount of pre-existing data, and data collection and processing to reduce noise is a time-consuming process. More crucially, data collection introduces uncertainty, depending on different means and methods (e.g., instrument manipulation or data analysis) [149]. These drawbacks limit the application of these methods for the sustainable development of nanomaterials. Therefore, researchers need to pay more attention to the disposal stage and purposefully collect data at this stage. Faced with the increasing number of new nanomaterials, high-throughput screening methods and ML are needed to quickly identify toxicity [148]. Reliable and useful data are especially scarce, thus a standardized data collection method is even more important.

The second challenge is data harmonization, which prevents cross-the-board analysis. Currently, most of these methods are used independently, and few materials are designed with a combination of LCA, RA, and ML. Specifically, LCA and RA of nanomaterials focus on different data perspectives, which makes the conclusions of the two approaches often contradictory and, therefore, difficult to use in combination [142]. Moreover, ML often involves integrating disparate data from multiple sources that differ in type, format, and semantics, making it difficult to integrate and use with other approaches [149]. As a result, LCA and RA evaluation data are rarely further used for ML to guide material design, due to the knowledge gap between different methodologies. Therefore, it is important to find a more appropriate strategy to balance the data outcomes of these approaches. Subsequently, toxicity factors and risk factors identified in RA and LCA can be standardized and used as environmental databases of materials for ML, guiding the further EHS design of nanomaterials.

The third challenge is the integration of experiments and AI tools. Although we have enabled the safe design and toxicity prediction of nanomaterials through AI tools on the surface, the effectiveness of these tools has not been fully empirically tested. The training set data used by AI tools are mostly collected from different kinds of literature and methods, and the data from a large number of experiments are rarely used, which causes a disconnect between actual experimental results and AI tools to a certain extent [150]. This makes the predictive accuracy of AI tools somewhat limited in practice. Hence, experiments and AI tools should complement each other. After using AI tools to achieve the safe design of nanomaterials, performance prediction, and toxicity prediction, it is necessary to verify the accuracy of the prediction results through experiments. The AI tools are then tweaked according to the difference between the predicted and observed experimental results. Only in this way can AI tools be truly implemented in the EHS-oriented development of nanomaterials.

In addition to the above challenges, we should also focus on the gap between theoretical research and the actual environment. The most frequent difference between research and environmental conditions is the nanomaterial concentration. On the one hand, whether for nano-remediation or nanotoxicology research, the concentration used is often higher than the actual environmental concentration to a certain extent. Although the use of high concentration is informative for the investigation of underpinning mechanisms, it is not reflective of the actual environment. On the other hand, the higher the concentration of the nanomaterials, the better the environmental repair effect within a certain range; however, the more severe the corresponding biological toxicity. Faced with the duality and the imbalance between the two research fields, despite attempts at intersecting, paradigms within each field are vastly different, hence progress is limited. In the future, we should try our best to gradually reduce the concentration of nanomaterials as far as feasible for practical remediation applications and assessment of environmental exposure toxicity.

In conclusion, interdisciplinary research continues to contribute to innovation in the field of science, and EHS-oriented development of nanomaterials will certainly flourish in the near future.

## CRediT authorship contribution statement

**Shuangyu Wu:** Data curation, Investigation, Visualization, Writing – original draft. **Jian Peng:** Data curation, Investigation, Visualization, Writing – original draft, Writing – review & editing. **Stephanie Ling Jie Lee:** Investigation, Writing – original draft, Writing – review & editing. **Xiaoqing Niu:** Investigation, Visualization. **Yue Jiang:** Visualization, Writing – review & editing. **Sijie Lin:** Conceptualization, Funding acquisition, Project administration, Supervision, Writing – review & editing.

## Declaration of competing interests

The authors declare no conflicts of interests.
